# Supplementary material for: Content-rich biological network constructed by mining PubMed abstracts
Source: BMC Bioinformatics. 2004 Oct 8;5:147. doi: 10.1186/1471-2105-5-147 (PMC528731; doi:10.1186/1471-2105-5-147)
Supplement: Additional File 2 — The original results of the above study (non-essential files are deleted to keep the file size under the limit set by BMC bioinformatics). [file 1471-2105-5-147-S2.bz2 › chilibotAdditionalFile2/dip40/326ID10638746E3625/html/TFRC_HFE.html]

 


 **TFRC** and **HFE** 
  
Found 145 abstracts in PubMed, retrieved 40.  
 

 What does Google say? 
 PDF only 
| .edu only 

---

**Interactive relationship** (e.g. stimulation, inhibition, etc)

**Inhibitory relationship**- The  **HFE**  protein binds to transferrin receptor  [ **TFRC** ]  1 TfR1  [ **TFRC** ]  in competition with transferrin, and in vitro, reduces cellular iron by reducing iron uptake.  Ref: 12429850 Proc Natl Acad Sci U S A, 2002
- Cells expressing a chimeric  **HFE**  protein with the cytoplasmic domain of the lysosomal protein, LAMP1, in place of its own  **HFE**  LAMP, show a decrease in the half life of the TfR  [ **TFRC** ]  implying that the interaction between  **HFE**  and TfR  [ **TFRC** ]  in endosomes targets the TfR  [ **TFRC** ]  to lysosomal compartments.  Ref: 12667138 Biochem J< MedlineTA>Biochem J, 2002
- We conclude that  **HFE**  has two mutually exclusive functions, binding to TfR1  [ **TFRC** ]  in competition with Tf, or inhibition of iron release.  Ref: 12429850 Proc Natl Acad Sci U S A, 2002
- This HCMV US2 mediated degradation of  **HFE**  leads to increased intracellular iron pools as indicated by reduced synthesis of TfR  [ **TFRC** ]  and increased ferritin synthesis.  Ref: 12456502 Blood, 2003
- To test the effect of  **HFE**  on lowering intracellular iron levels independently of its association with TfR  [ **TFRC** ] , a mutated  **HFE**  fW81AHFE that shows greatly reduced affinity for the TfR  [ **TFRC** ]  was transfected into tetracycline controlled transactivator HeLa cells.  Ref: 12874382 Proc Natl Acad Sci U S AProc Natl Acad Sci U S A, 2003
- However, cells expressing  **HFE**  still showed a decrease in Tf mediated iron uptake at concentrations of Tf sufficient to dissociate  **HFE**  from the TfR  [ **TFRC** ] .  Ref: 12874382 Proc Natl Acad Sci U S AProc Natl Acad Sci U S A, 2003
**Neutral relationship**- At variance with  **TFRC** , interactions between TFR2 and  **HFE**  do not occur, at least in their soluble forms.  Ref: 12547237 Blood Cells Mol Dis< MedlineTA>Blood Cells Mol Dis, 2003
- The interaction between TfR  [ **TFRC** ]  and  **HFE**  LAMP was confirmed by immunoprecipitation, in addition to immunofluorescence studies.  Ref: 12667138 Biochem J< MedlineTA>Biochem J, 2003
- Targeted mutagenesis of the murine transferrin receptor  [ **TFRC** ]  2 gene produces hemochromatosis  [ **HFE** ] .  Ref: 12134060 Proc Natl Acad Sci U S A, 2002
- Recent studies have revealed that the wild type hemochromatosis  [ **HFE** ]  protein  **HFE**  interacts with the transferrin receptor  [ **TFRC** ]  TfR  [ **TFRC** ]  and modulates TfR  [ **TFRC** ]  mediated iron uptake by cells.  Ref: 12791823 J Nucl Med, 2003
- The hereditary hemochromatosis protein  [ **HFE** ]   **HFE**  is known to complex with the transferrin receptor  [ **TFRC** ] .  Ref: 11940510 Am J Physiol Cell Physiol, 2002
- Recent reports suggest that the mutations of transferrin receptor  [ **TFRC** ]  2 TfR2 gene may be involved in non  **HFE**  hemochromatosis  [ **HFE** ] .  Ref: 12809944 Hepatol Res< MedlineTA>Hepatol Res, 2002
- Mutations affecting the TFR  [ **TFRC** ]  2 gene led to hemochromatosis  [ **HFE** ]  type 3, a form of inherited iron overload.  Ref: 12393650 Blood, 2002
- Wild type  **HFE**  protein binds to the transferrin receptor  [ **TFRC** ] , and by an undefined mechanism the enterocyte is programmed to absorb an amount of dietary iron precisely matched to the body s needs.  Ref: 12382198 Semin Hematol, 2002
- **HFE**  protein was recently found to coprecipitate with the transferrin receptor  [ **TFRC** ]  and to affect the reaction between transferrin and the transferrin receptor  [ **TFRC** ] .  Ref: 11941597 Zhonghua Yi Xue Yi Chuan Xue Za Zhi, 2002
- The proposed role for  **HFE**  is its part in the regulation of the interaction of the transferrin receptor  [ **TFRC** ]  with transferrin.  Ref: 11941597 Zhonghua Yi Xue Yi Chuan Xue Za Zhi, 2002
- Homozygous defects in a recently identified gene encoding transferrin receptor  [ **TFRC** ]  2 lead to iron overload hemochromatosis  [ **HFE** ]  type 3 with symptoms similar to those seen in patients with  **HFE**  associated hereditary hemochromatosis  [ **HFE** ]  hemochromatosis  [ **HFE** ]  type 1.  Ref: 12382200 Semin Hematol, 2002
- Although the  **HFE**  protein appears to modulate the function of the transferrin receptor  [ **TFRC** ]  in vitro, its precise role in vivo remains obscure.  Ref: 12657433 Trends Mol Med, 2003
- Less common non  **HFE**  related forms of hemochromatosis  [ **HFE** ]  have been reported and are caused by mutations in the transferrin receptor  [ **TFRC** ]  2 gene and in a gene localized to chromosome 1q.  Ref: 12091366 Blood, 2002
- This protein regulates transferrin receptor  [ **TFRC** ]  mediated iron uptake and mutations in its gene cause hereditary iron overload hemochromatosis  [ **HFE** ] .  Ref: 12461085 J Exp Med, 2002
- **HFE** , the protein mutated in Hereditary Hemochromatosis  [ **HFE** ]  Type 1, is known to interact with the transferrin receptor  [ **TFRC** ]  TfR  [ **TFRC** ]  on the cell surface and during endocytosis Gross, C.N. et al.  Ref: 12667138 Biochem J< MedlineTA>Biochem J, 2002
- **HFE**  is a nonclassical class I molecule that associates with beta 2 microglobulin beta 2m and with the transferrin receptor  [ **TFRC** ] .  Ref: 12456502 Blood, 2003
- Haemochromatosis  [ **HFE** ]  type 3 is linked to a locus on 7q22 and is due to mutations in the transferrin receptor  [ **TFRC** ]  2.  Ref: 12401307 Best Pract Res Clin Haematol, 2002
- Evidence for the interaction of the hereditary hemochromatosis protein  [ **HFE** ] ,  **HFE** , with transferrin receptor  [ **TFRC** ]  in endocytic compartments.  Ref: 12667138 Biochem J< MedlineTA>Biochem J, 2002
- Type 3 hemochromatosis  [ **HFE** ] , recently described in 4 families, is linked to mutations of the gene encoding transferrin receptor  [ **TFRC** ]  2 on chromosome 7q22.  Ref: 11984516 Gastroenterology, 2002
- CONCLUSION Our studies suggest that expression of wild type  **HFE**  in cells produces a decrease in 67 Ga uptake due to a reduction in available Tf binding sites for 67 Ga Tf on the TfR  [ **TFRC** ] .  Ref: 12791823 J Nucl Med, 2003
- Consistent with earlier observations in other cell lines, the expression of wild type or H63D, but not C282Y,  **HFE**  induces an apparent iron deficient phenotype, manifested in the activation of iron regulatory protein and concomitant increase in transferrin receptor  [ **TFRC** ]  levels and decrease in ferritin content.  Ref: 12464008 Biochem J, 2003

**Non-interactive relationship** (e.g. studied together, co-existance, homology, etc.)

- CONCLUSIONS Our data support the role of the transferrin receptor  [ **TFRC** ]  2 gene in hemochromatosis  [ **HFE** ]  type 3 as well as its critical involvement in the maintenance of iron homeostasis in humans.  Ref: 11984516 Gastroenterology, 2002
- The function of  **HFE**  protein is unknown, but it is hypothesized that it acts in association with beta 2 microglobulin and transferrin receptor  [ **TFRC** ]  1 to regulate iron uptake from plasma transferrin by the duodenum, the proposed mechanism by which body iron levels are sensed.  Ref: 11943867 Proc Natl Acad Sci U S A, 2002
- A recent study suggests that  **HFE**  controls the recycling rate of the transferrin receptor  [ **TFRC** ]  and thereby ultimately controls the iron status of the enterocyte.  Ref: 12030278 Nutr Rev, 2002
- METHODS The expression and localization of  **HFE** , TfR1  [ **TFRC** ]  and TfR2 proteins in human platelets were examined using Western blotting and immunocytochemistry.  Ref: 12656741 Eur J Haematol, 2003
- The aim of this study was to assess the role of hepatic iron,  **HFE**  and TfR1  [ **TFRC** ]  variations on development and progression of chronic hepatitis C infection.Methods We studied 119 consecutive patients with chronic hepatitis C, correlating clinical, laboratory, histopathological, and genetic data.  Ref: 12445428 J Hepatol, 2002
- Recently a second transferrin receptor  [ **TFRC** ]  TFR2 was discovered, and a previously uncharacterized type of hemochromatosis  [ **HFE** ]  HH type 3 was identified in humans carrying mutations in the TFR2 gene.  Ref: 12134060 Proc Natl Acad Sci U S A, 2002
- **HFE**  accumulates in transferrin containing endosomes, and its overexpression in human cell lines correlates with decreased transferrin receptor  [ **TFRC** ]  TFR  [ **TFRC** ]  mediated iron uptake and decreased intracellular iron pools.  Ref: 12456502 Blood, 2003
- Clinical and pathologic findings in hemochromatosis  [ **HFE** ]  type 3 due to a novel mutation in transferrin receptor  [ **TFRC** ]  2 gene.  Ref: 11984516 Gastroenterology, 2002
- These results indicate that the association of  **HFE**  with TfR  [ **TFRC** ]  is not essential for its ability to lower intracellular iron stores.  Ref: 12874382 Proc Natl Acad Sci U S AProc Natl Acad Sci U S A, 2002
- Coimmunoprecipitation studies using solubilized cell extracts demonstrated that transferrin Tf competed with  **HFE**  for binding to the transferrin receptor  [ **TFRC** ]  TfR  [ **TFRC** ]  similar to previous in vitro studies using soluble truncated forms of  **HFE**  and the TfR  [ **TFRC** ] .  Ref: 12874382 Proc Natl Acad Sci U S AProc Natl Acad Sci U S A, 2002
- **HFE** , the protein defective in hereditary hemochromatosis  [ **HFE** ] , and transferrin receptor  [ **TFRC** ]  2 TfR2 are two novel protein candidates that could be involved in mechanisms of iron transport across the platelet plasma membrane.  Ref: 12656741 Eur J Haematol, 2003
- Addition of transferrin Tf to  **HFE**  LAMP expressing cells competes with  **HFE**  for binding to the TfR  [ **TFRC** ]  thereby increasing the half life of TfR  [ **TFRC** ]  and confirming that the  **HFE**  LAMP TfR  [ **TFRC** ]  complex is reaching the cell surface prior to entering the endosomal vesicles and trafficking to the lysosome.  Ref: 12667138 Biochem J< MedlineTA>Biochem J, 2003
- AIMS To investigate this issue, we examined concomitant variations in duodenal cytochrome b Dcytb, divalent metal transporter 1 DMT1, ferroportin 1 FPN1, hephaestin, stimulator of Fe transport SFT,  **HFE** , and transferrin receptor  [ **TFRC** ]  1 TfR1  [ **TFRC** ]  transcripts in response to different dietary iron contents in the four mouse strains C57BL 6, DBA 2, CBA, and 129 Sv.  Ref: 12377801 Gut, 2002
- Iron and  **HFE**  or TfR1  [ **TFRC** ]  mutations as comorbid factors for development and progression of chronic hepatitis C.  Ref: 12445428 J Hepatol, 2002
- Such changes are detected by transferrin receptor  [ **TFRC** ]  2 and the  **HFE**  transferrin receptor  [ **TFRC** ]  1 complex.  Ref: 12737947 Blood Cells Mol Dis, 2003
- Duodenal expression of  **HFE**  and TfR2 but not TfR1  [ **TFRC** ]  in wild type mice and humans was restricted to crypt cells, in which they co localized.  Ref: 12704209 J Histochem Cytochem, 2003
- Transferrin receptor  [ **TFRC** ]  2 TfR2 and  **HFE**  mutational analysis in non C282Y iron overload identification of a novel TfR2 mutation.  Ref: 12130528 Blood, 2002
- TfR1  [ **TFRC** ]  polymorphisms bore no detectable relation to disease severity or response to therapy.Conclusions Hepatic iron and  **HFE**  mutations are comorbid factors that increase development and progression of chronic hepatitis C.  Ref: 12445428 J Hepatol, 2002
- Can defects in transferrin receptor  [ **TFRC** ]  2 and hereditary hemochromatosis  [ **HFE** ]  genes account for iron overload in HbH disease ? < ArticleTitle>.  Ref: 12667993 Blood Cells Mol Dis< MedlineTA>Blood Cells Mol Dis, 2002
- Human transferrin receptor  [ **TFRC** ]  2 is 45% identical with transferrin receptor  [ **TFRC** ]  1 in the extracellular domain, but contains no iron responsive element in its mRNA and is apparently not regulated by intracellular iron concentration nor by interaction with  **HFE** .  Ref: 12531241 Int J Biochem Cell Biol, 2003
- The transferrin receptor  [ **TFRC** ]  2 TFR2 and hereditary hemochromatosis  [ **HFE** ]   **HFE**  genes were examined to see if inheritance of these gene defects may be a possible cause of iron overload in 45 HbH patients.  Ref: 12667993 Blood Cells Mol Dis< MedlineTA>Blood Cells Mol Dis, 2003
- The presence of TfR2 and absence of TfR1  [ **TFRC** ]  suggests that  **HFE**  may serve a different function in platelets compared with the other  **HFE**  positive cell types, e.g. enterocytes, macrophages and syncytiotrophoblasts.  Ref: 12656741 Eur J Haematol, 2003
- In addition, we find that the HH associated mutant H41D has lost the ability to inhibit iron release despite binding to TfR1  [ **TFRC** ]  as well as wild type  **HFE** .  Ref: 12429850 Proc Natl Acad Sci U S A, 2002
- Mutations or polymorphisms in the  **HFE**  gene or the TfR1  [ **TFRC** ]  gene may influence the accumulation of iron in the liver or other tissues or may influence chronic viral hepatitis apart from effects on iron homeostasis.  Ref: 12445428 J Hepatol, 2002
- Human platelets express hemochromatosis  [ **HFE** ]  protein  **HFE**  and transferrin receptor  [ **TFRC** ]  2.  Ref: 12656741 Eur J Haematol, 2003
- Co localization of the mammalian hemochromatosis  [ **HFE** ]  gene product  **HFE**  and a newly identified transferrin receptor  [ **TFRC** ]  TfR2 in intestinal tissue and cells.  Ref: 12704209 J Histochem Cytochem, 2003
- The following candidate genes have been examined in detail to determine whether polymorphisms in them may be responsible for this variation transferrin, transferrin receptor  [ **TFRC** ]  1, transferrin receptor  [ **TFRC** ]  2, ferritin L, ferritin H, IRP1, IRP2,  **HFE** , beta 2 microglobulin, mobilferrin calreticulin, ceruloplasmin, ferroportin, NRAMP1, NRAMP2 DMT1, haptoglobin, heme oxygenase 1, heme oxygenase 2, hepcidin, USF2, ZIRTL, duodenal cytochrome b ferric reductase DCYTB, TNFalpha, keratin 8, and keratin 18.  Ref: 12547238 Blood Cells Mol Dis< MedlineTA>Blood Cells Mol Dis, 2003
- The results indicate that  **HFE**  can lower intracellular iron levels independently of its interaction with the TfR  [ **TFRC** ] .  Ref: 12874382 Proc Natl Acad Sci U S AProc Natl Acad Sci U S A, 2003
- At concentrations of Tf approaching those found in the blood, no differences in Tf binding to cells were detected, which is consistent with the lower binding constant of  **HFE**  for TfR  [ **TFRC** ]  versus Tf.  Ref: 12874382 Proc Natl Acad Sci U S AProc Natl Acad Sci U S A, 2003
- In iron overload, TfR1  [ **TFRC** ]  expression in the duodenum differed according to the presence or absence of the C282Y mutation in the  **HFE**  gene, being increased in  **HFE**  related hemochromatosis  [ **HFE** ]  and similar to controls in non  **HFE**  related iron overload.  Ref: 12547240 Blood Cells Mol Dis< MedlineTA>Blood Cells Mol Dis, 2003
- The function of  **HFE**  protein is unknown, but the available evidence suggests that it acts in association with beta2 microglobulin and transferrin receptor  [ **TFRC** ]  1 to regulate iron uptake from plasma transferrin by the duodenum, the proposed mechanism by which body iron levels are sensed.  Ref: 12416729 Int J Hematol, 2002
- These results raise the possibility that interaction of  **HFE**  and TfR  [ **TFRC** ]  in intracellular vesicles may play an important role in determining the function of  **HFE**  in iron homeostasis, which is still unknown.  Ref: 12667138 Biochem J< MedlineTA>Biochem J, 2002
- Hence, the availability of plasma transferrin bound iron and the expression and function of transferrin receptors TfR1  [ **TFRC** ] ,  **HFE**  and DMT1 should all contribute to the absorptive capacity of villus enterocytes.  Ref: 12547229 Blood Cells Mol Dis< MedlineTA>Blood Cells Mol Dis, 2002
- Strain to strain differences were observed not only in serum transferrin saturations, with C57BL 6 mice having the lowest values, but also in hepatic iron stores and in duodenal mRNA expression of Dcytb, DMT1, FPN1, hephaestin,  **HFE** , and TfR1  [ **TFRC** ] .  Ref: 12377801 Gut, 2002
- Progress in our understanding of how  **HFE**  regulates the absorption of dietary iron has been slow, but much can be learnt from the study of the rare instances of haemochromatosis  [ **HFE** ]  that involve mutations in newly identified iron metabolism genes, such as TFR2 a transferrin receptor  [ **TFRC** ]  isoform and ferroportin1 Ireg1 mtp1 an intestinal iron transporter.  Ref: 12457803 Lancet, 2002
- These aspects of the regulation and mechanism of iron absorption were investigated in genetically normal rats and mice, and in Belgrade anemic b b rats and  **HFE**  knockout mice.In most experiments the function of the TfR1  [ **TFRC** ]  was assessed by the uptake of radiolabeled transferrin bound iron given intravenously.  Ref: 12547229 Blood Cells Mol Dis< MedlineTA>Blood Cells Mol Dis, 2002
- Mutations in the  **HFE**  gene and a newly identified second transferrin receptor  [ **TFRC** ]  gene, TfR2, cause hemochromatosis  [ **HFE** ] .  Ref: 12704209 J Histochem Cytochem, 2003
- Hemochromatosis  [ **HFE** ]  due to mutations in transferrin receptor  [ **TFRC** ]  2.  Ref: 12547237 Blood Cells Mol Dis< MedlineTA>Blood Cells Mol Dis, 2003
